# Timing of donor lymphocyte infusion after relapse following allogeneic hematopoietic cell transplantation: a retrospective single-center analysis

**DOI:** 10.1007/s00277-026-07090-1

**Published:** 2026-05-30

**Authors:** Alexander C. Angleitner, Markus Maulhardt, Hannes Treiber, Justin Hasenkamp, Judith Büntzel, Gerald G. Wulf

**Affiliations:** Department of Hematology and Oncology, University Medical Hospital Goettingen, Robert Koch-Straße 40, +49 551 -3962051, 37075 Goettingen, Germany

**Keywords:** Acute leukemia, Myelodysplastic Syndrome, Relapse, Allogeneic hematopoietic cell transplantation, Donor lymphocyte infusion, Minimal residual disease

## Abstract

Donor lymphocyte infusions (DLI) are an important strategy for managing relapse after allogeneic hematopoietic stem cell transplantation (allo-HCT), yet data on the impact of surveillance frequency and DLI timing remain limited. We retrospectively analyzed 83 adults with acute leukemia or MDS who received DLI after relapse between 2010 and 2022. Monthly molecular monitoring with donor chimerism and genetic markers enabled classification of preemptive DLI at molecular relapse and therapeutic DLI at overt hematologic relapse. Overall survival (OS) was assessed using Kaplan–Meier estimates and Cox regression. Therapeutic DLI was associated with inferior OS compared with preemptive DLI (HR = 0.15 in multivariable analysis, p < 0.001); median OS was 0.47 years in the therapeutic group, while it was not reached after preemptive DLI. GvHD after DLI was associated with better OS (HR = 0.28), consistent with graft-versus-leukemia effects. A longer interval between allo-HCT and relapse also predicted improved survival (HR = 0.78). Delivering DLI at molecular rather than hematologic relapse improved survival, suggesting that close MRD-based surveillance enables earlier detection, timely immunologic intervention, and may improve post-relapse outcomes.

## Introduction

 Relapse remains the most common cause of treatment failure after allogeneic hematopoietic stem cell transplantation (allo-HCT) in patients with acute leukemia and myelodysplastic syndromes (MDS) [1]. Donor lymphocyte infusion (DLI) is a commonly employed post-relapse strategy [1, 2] aimed at enhancing the graft-versus-leukemia (GvL) effect [3–6]. Although graft-versus-host disease (GvHD) is the most common toxicity associated with DLI, mortality directly attributable to GvHD is rare, accounting for approximately 6% of deaths [7].

Response rates to DLI vary by disease [8] with response rates in acute myeloid leukemia (AML; 30–40%) [9], acute lymphoblastic leukemia (ALL; up to 15%) [10], and MDS (up to 21%) [11].

DLI are administered for three distinct indications: prophylactic, preemptive, and therapeutic [2]. Prophylactic DLI are delivered at predefined intervals in high-risk patients [12, 13] without evidence of the underlying hematological disease [14]. Preemptive DLI are initiated in response to early relapse indicators, such as minimal residual disease (MRD) or loss of complete donor chimerism (LOC) [15, 16]. Therapeutic DLI are administered against overt hematologic relapse [2].

At our center, intensified MRD monitoring at monthly intervals has been implemented to facilitate early relapse detection and timely intervention. Nevertheless, it remains uncertain whether such close surveillance translates into improved survival, which patient subgroups benefit most from early intervention, and how DLI-related GvHD interacts with relapse characteristics. Here, we present a single-center analysis incorporating frequent MRD monitoring, clearly defined relapse categories, and systematic subgroup evaluation.

## Methods

This retrospective study was conducted in accordance with the Declaration of Helsinki and approved by the institutional ethics committee (approval number 9/8/25).

**Fig. 1:**
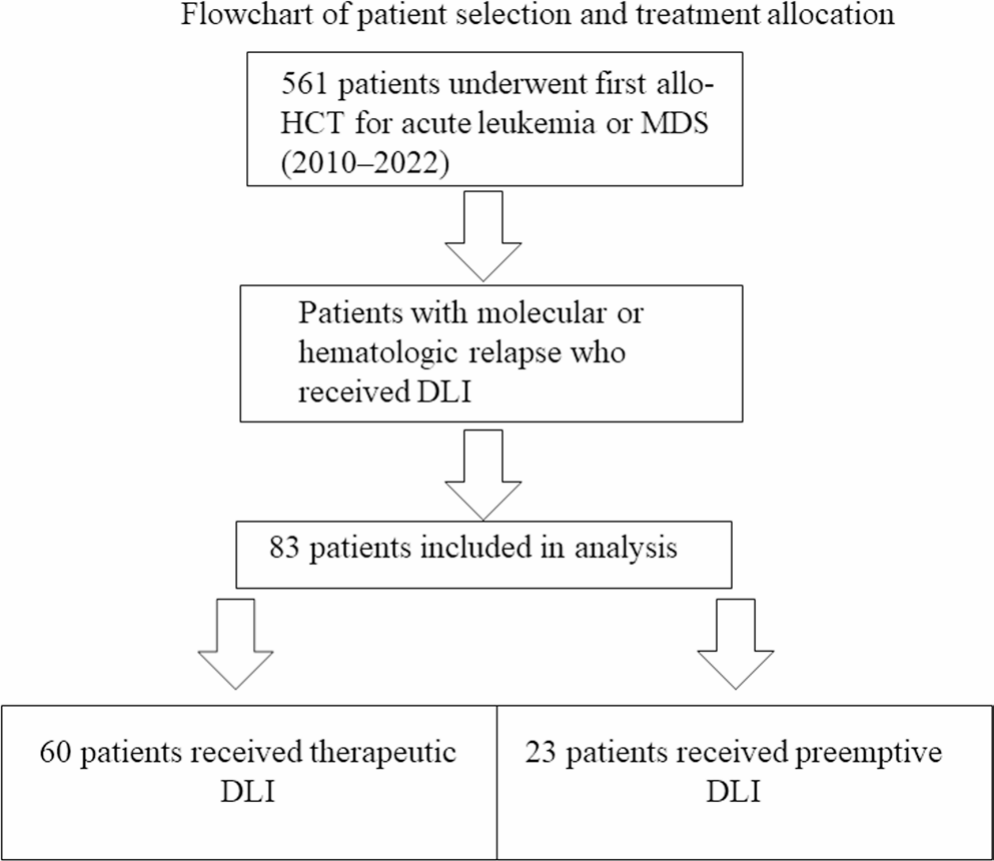
Flowchart of patient selection and treatment allocation

The analysis included 83 patients (≥ 18 years) who received DLI for molecular or hematological relapse following allo-HCT for acute leukemia or MDS. All patients underwent allo-HCT between January 2010 and December 2022 (Fig. 1). Demographic and clinical variables included age, sex, disease type, cytogenetic profile, conditioning regimen, time to relapse, DLI details, and occurrence of GvHD (graded according to Harris classification [17]). Chronic GvHD was graded according to NIH consensus criteria [18].

Preemptive DLI were defined as administration at the time of molecular relapse in clinically stable patients without overt hematologic deterioration. Molecular relapse was identified by MRD positivity assessed by molecular analysis using next-generation sequencing (NGS) or fluorescence in situ hybridization (FISH)–based markers, or by a LOC exceeding 5%. Applied MRD thresholds reflected routine clinical practice at our center during the study period.

Following allo-HCT, all patients achieved full donor chimerism and were monitored for molecular relapse. DLI treatment was triggered by molecular evidence of relapse, defined as decreasing donor chimerism or increasing disease-specific molecular markers.

Donor chimerism was measured at total peripheral blood (PB) DNA level, applying the ABI 3500 GeneScan Dx and GeneScanc ABI 3130 system (Applied Biosystems, Foster City, CA, USA). Measurement of MRD was performed by NGS analysis of 53 myeloid genetic disease alteration, using the QIAseq Targeted DNA custom panel (QIAGEN, Hilden, Germany). High-throughput sequencing was conducted on a MiSeq or MiniSeq platform (Illumina, San Diego, CA, USA). FISH analysis was performed on 200 CD34-enriched PB cells, targeting case-specific cytogenetic alterations. FISH was carried out using commercially available locus-specific probes (e.g., *BCR-ABL1*, *KMT2A*, *TP53*) from Abbott/Vysis and MetaSystems according to the manufacturers’ protocols.

Therapeutic DLI were defined as administration at overt clinical or hematological disease evidence. Patients who experienced both molecular and clinical relapse were categorized as receiving therapeutic DLI. At our center, monthly molecular monitoring (MRD and/or donor chimerism) is routinely performed during the first year after allo-HCT. During the second and third year after transplantation, surveillance is typically conducted every three months. Beyond three years, no standardized monitoring protocol is applied, and molecular testing is performed based on clinical indications such as symptoms suggestive of relapse or other clinical findings. This approach allowed detection of later relapses occurring beyond the first post-transplant year.

DLI were administered in the absence of active GvHD, typically at monthly intervals with stepwise dose escalation starting at 0.5 × 10^6 CD3 + cells/kg and increasing to 5 × 10^6, and up to 1 × 10^7 cells/kg body weight, with a maximum of five applications. The actual number of DLI infusions depended on donor lymphocyte availability, treatment tolerance, and clinical response. DLI administration was discontinued in cases of disease progression, lack of response, or treatment-related toxicity.

The interval between relapse detection and DLI administration reflected routine clinical practice and included logistical and clinical factors such as donor availability, scheduling of lymphocyte collection, cell procurement and processing, prior or ongoing graft-versus-host disease, disease kinetics, and individualized treatment decisions. In selected cases, bridging therapy or clinical stabilization was required prior to DLI administration.

If patients developed GvHD greater than grade I during DLI treatment, further DLI administration was discontinued. In some cases, DLI were combined with additional therapies such as hypomethylating agents according to clinical judgment. In selected patients, DLI were also used as part of a treatment strategy prior to a second allo-HCT.

Overall survival (OS) was defined from the date of first DLI to death from any cause or last follow-up. Patients who subsequently underwent a second allo-HCT were not censored at the time of transplantation but were followed until death or last contact in order to reflect real-world post-relapse treatment courses and to avoid treatment-dependent censoring. Kaplan-Meier estimates [19] and log-rank tests were used to compare survival distributions. Cox proportional hazards regression was applied for univariate and multivariate analysis. Variables with a p-value < 0.20 in univariate analysis were entered into the multivariate Cox regression model. Statistical analyses were conducted using Python 3.11 (Python Software Foundation, https://www.python.org) with the lifelines package (v0.27.0) for survival analysis, pandas (v1.5.3) for data handling, and matplotlib (v3.6.2) for visualization.

Artificial intelligence–based tools were used to support language editing and code refinement; all analyses, code, and interpretations were fully reviewed, validated, and approved by the authors, who take full responsibility for the analytical workflow.

## Results

Among the 83 patients receiving DLI after relapse post allo-HCT, 32 (39%) were female and 51 (61%) male. The median age at first DLI infusion was 61.3 years (IQR 52.0-66.3). The underlying diagnoses were AML (*n* = 45, 54%), secondary AML (sAML) (*n* = 18, 22%), MDS (*n* = 13, 16%), and ALL (*n* = 7, 8%). Further clinical characteristics are summarized in Table 1.

**Table 1:** Baseline clinical characteristics at the time of first donor lymphocyte infusion

Variable	Overall cohort (*n* = 83)	Therapeutic DLI (*n* = 60)	Preemptive DLI (*n* = 23)
Patient characteristics at relapse			
Age at intervention in years, median [IQR]	61.33 [52.00-66.30]	59.78 [49.76–65.67]	60.38 [54.28–64.90]
ECOG at intervention, median [IQR]	1 [0–1]	1 [0–1]	1 [0–1]
Deaths, n (%)	52 (63)	49 (82)	3 (13)
Sex, n (%)			
Male	51 (61)	33 (55)	18 (78)
Female	32 (39)	27 (45)	5 (22)
Diagnosis, n (%)			
AML	45 (54)	30 (50)	15 (65)
sAML	18 (22)	15 (25)	3 (13)
MDS	13 (16)	11 (18)	2 (9)
ALL	7 (8)	4 (7)	3 (13)
Disease status prior to allo-HCT			
Complete remission	33 (40)	20 (33)	13 (57)
Partial remission	11 (13)	8 (13)	3 (13)
Active disease /relapse	16 (19)	14 (23)	2 (9)
Unknown disease status	12 (14)	8 (13)	4 (17)
Time from allo-HCT to relapse (years), median [IQR]	0.84 [0.47–2.07]	0.79 [0.48–2.08]	1.01 [0.46–2.03]
Donor type at allo-HCT, n (%)			
Matched related	17 (20)	10 (17)	7 (30)
Matched unrelated	54 (65)	40 (67)	14 (61)
Mismatched unrelated	11 (13)	10 (17)	1 (4)
Haploidentical	1 (1)		1 (4)
Conditioning intensity (1st allo), n (%)			
Myeloablative	38 (46)	22 (37)	16 (70)
Reduced intensity	45 (54)	38 (63)	7 (30)
Treatment-specific characteristics			
Time from relapse to DLI (days), median [IQR]	44 [35–78]	42.5 [35–68]	47 [35–102]
DLI number, median [IQR]	3 [2–4]	3 [2–4]	3 [3–4]
GvHD after DLI n (%)	22 (27)	9 (15)	13 (57)
Acute GvHD (Harris)			
Grade I	6 (7)	2 (3)	4 (17)
Grade II	5 (6)	2 (2)	3 (13)
Grade III	6 (7)	2 (3)	4 (17)
Grade IV	2 (2)	1 (2)	1 (4)
Chronic GvHD (NIH Consensus), n (%)			
mild	2 (2)	1 (2)	1 (4)
moderate	5 (6)	4 (8)	1 (4)
Patients who received second allo-HCT after DLI, n (%)	12 (14)	9 (15)	3 (13)

Abbreviations: *AML* acute myeloid leukemia,= *sAML* secondary *AML*, *MDS* myelodysplastic syndrome, *ALL* acute lymphoblastic leukemia, *allo-HCT*, allogeneic hematopoietic stem cell transplantation, *DLI* donor lymphocyte infusion, *GvHD* graft-versus-host disease, *ECOG* Eastern Cooperative Oncology Group. *CR* includes *CRi* and cytomorphological remission. Active disease includes refractory disease, relapse, or progressive disease prior to transplantation. Continuous variables are reported as median (interquartile range [IQR], 25th–75th percentile). Reported IQR values do not represent minimum–maximum ranges

**Fig. 2:**
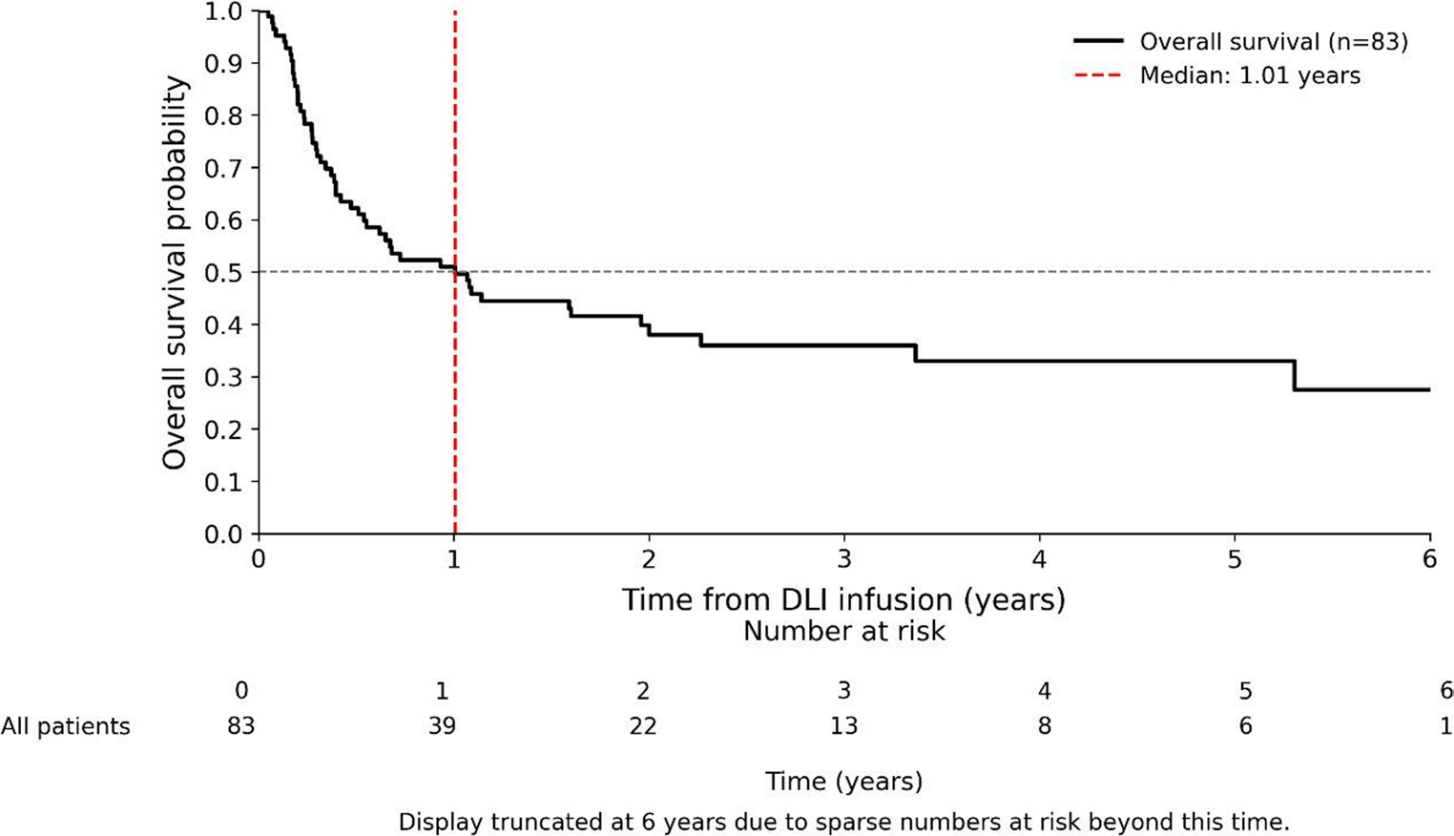
Kaplan–Meier estimates of overall survival (OS) from the time of first donor lymphocyte infusion (DLI)

The median OS from DLI infusion was 1.01 years (95% CI, 0.47–5.31) (Fig. 2). The median follow-up, calculated by reverse Kaplan–Meier, was 2.55 years. Estimated OS rates at 1, 2, 3, and 5 years were 49.7% (95% CI, 38.4–60.0), 38.0% (27.1–48.8), 36.0% (25.1–47.0), and 28.0% (15.0–41.7), respectively. The survival curve plateaued at ~ 28% after the last observed event (5.31 years). The median interval from relapse to first DLI was 44 days (IQR: 35–78), and the median number of DLI infusions administered was 3 (IQR: 2–4). At the time of analysis, 52 patients (63%) had died, while 31 (37%) were censored. Causes of death included disease relapse or progression (32/52), sepsis (11/52), intracerebral hemorrhage (2/52), and GvHD (2/52); in five cases the cause was unknown.

**Fig. 3:**
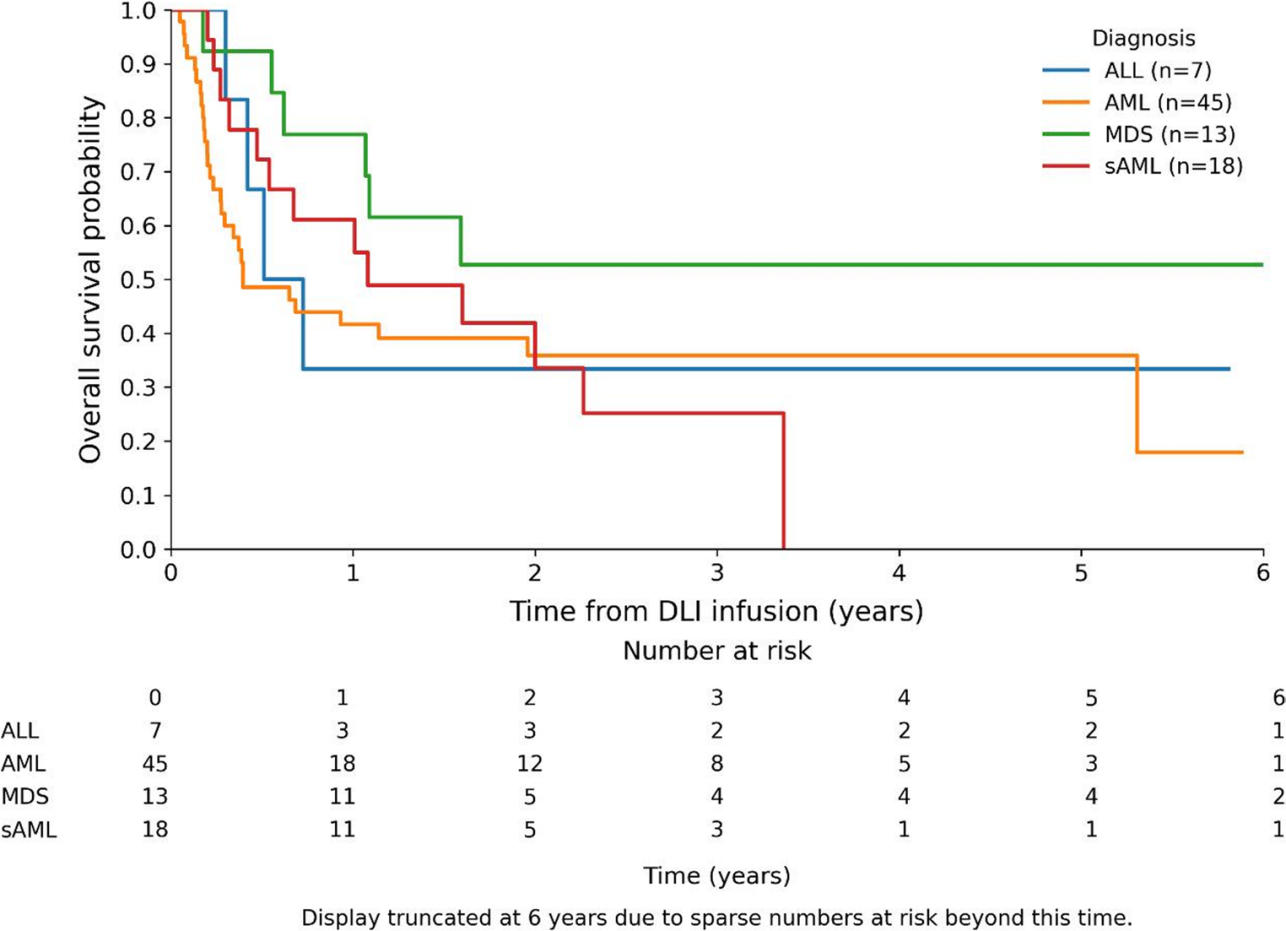
Kaplan Meier estimates of overall survival stratified by diagnosis

OS did not significantly differ across diagnostic subgroups (AML, sAML, MDS, ALL) after Bonferroni-adjusted pairwise log-rank tests (Fig. 3).

**Fig. 4:**
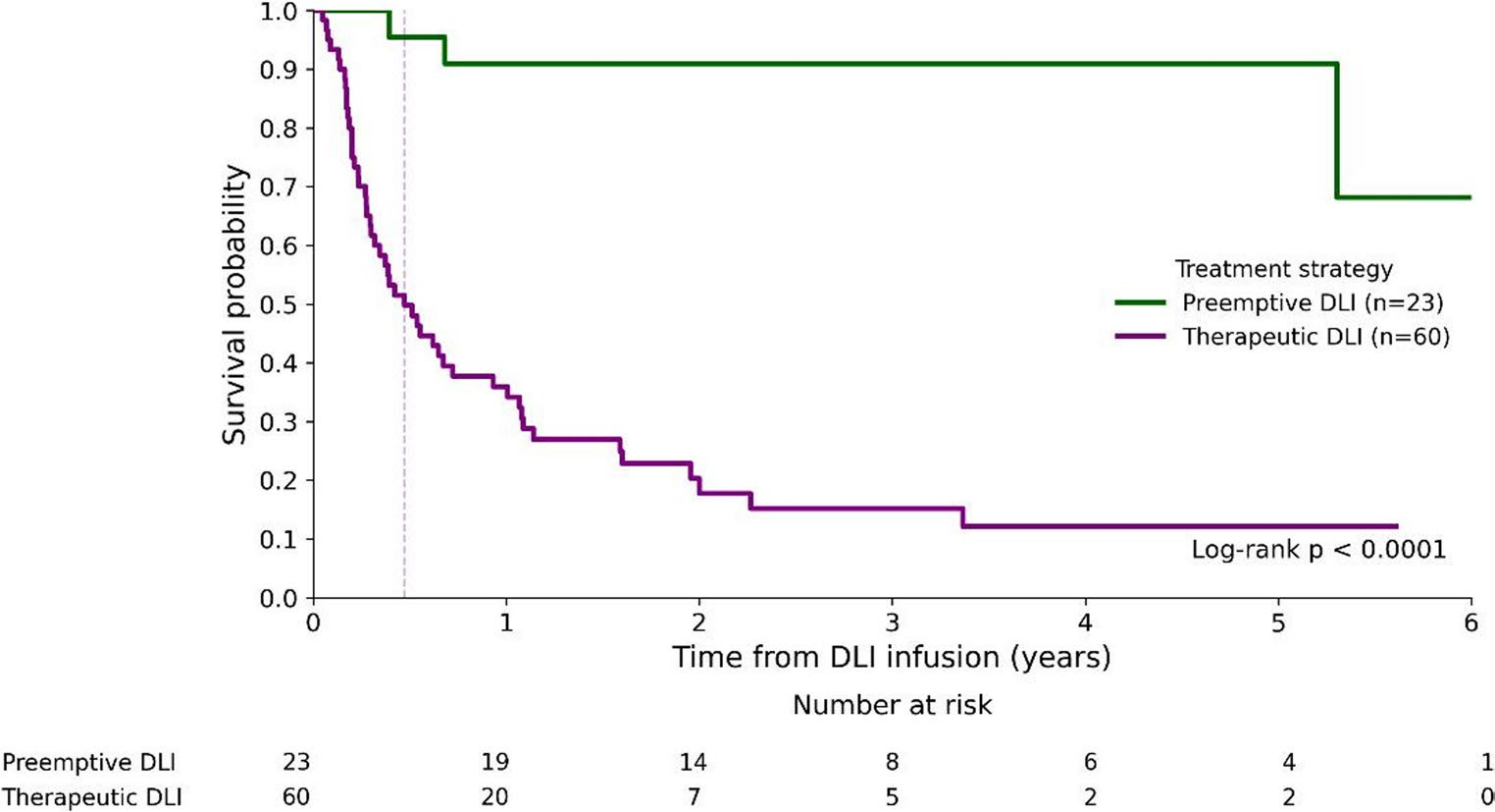
Kaplan–Meier estimates of overall survival comparing preemptive DLI administered at molecular relapse and therapeutic DLI administered at overt hematologic relapse

Baseline disease distribution was comparable between groups. AML was the underlying diagnosis in 50% of patients receiving therapeutic DLI and in 65% of those receiving preemptive DLI. Median OS was 0.47 years (95% CI, 0.29–0.72) in the therapeutic group, whereas it was not reached in the preemptive group (95% CI, ≥5.31 years; log-rank p < 0.001; Figure 4).

Preemptive DLI (HR=0.08, 95% CI: 0.02-0.26, p < 0.001) and the occurrence of GvHD following DLI (HR =0.15, 95% CI: 0.06–0.37; p < 0.001) were significantly associated with favorable OS in univariate analysis (Table 2).

**Table 2:** Univariate Cox Regression for overall survival after DLI infusion

Baseline items	HR (95% CI)	*p*-value
Univariate analysis (83/83)		
Age at DLI infusion	1.01 (0.98–1.03)	0.619
Time from allo-HCT to relapse (years)	0.87 (0.72–1.06)	0.160
Time from Relapse to DLI (days)	1.00 (0.99–1.00)	0.307
Preemptive DLI (vs. therapeutic DLI)	0.08 (0.02–0.26)	< 0.001
GvHD after DLI (vs. no GvHD)	0.15 (0.06–0.37)	< 0.001

Hazard ratios < 1 indicate improved survival. Reference categories were therapeutic *DLI* and absence of *GvHD*

In the multivariate Cox regression model (Table 3), preemptive DLI remained an independent predictor of improved OS (HR = 0.15, 95% CI: 0.06–0.36; p < 0.001). The occurrence of GvHD (HR = 0.28, 95% CI: 0.13–0.63; p = 0.002) and a longer interval from allo-HCT to relapse (HR = 0.81, 95% CI: 0.69–0.96; p = 0.015) also retained statistical significance. Other covariates, including age were not significant. 

**Table 3:** Multivariate Cox Regression for overall survival after DLI infusion

Baseline items	HR (95% CI)	*p*-value
Multivariate analysis (83/83)		
Age at DLI infusion	1.02 (0.99–1.04)	0.144
Time from allo-HCT to relapse (years)	0.81 (0.69–0.96)	0.015
Preemptive DLI (vs. therapeutic DLI)	0.15 (0.06–0.36)	< 0.001
GvHD after DLI (vs. no GvHD)	0.28 (0.13–0.63)	0.002

Hazard ratios < 1 indicate improved survival. Reference categories were therapeutic *DLI* and absence of *GvHD*

These findings confirm the independent prognostic impact of preemptive DLI and post-DLI GvHD, both of which remained strongly associated with improved OS after multivariable adjustment, underscoring the importance of timely intervention and effective immunologic response in the post-relapse setting.

## Discussion 

In this study, patients who received preemptive DLI at the stage of molecular relapse had markedly improved survival compared to those receiving therapeutic DLI. This is consistent with prior studies [20–22] and supports an association between earlier immunologic intervention and improved outcomes. The rationale of this approach was to detect disease recurrence at the stage of MRD positivity, thereby permitting consideration of preemptive DLI within routine clinical decision-making.

Among 60 patients who received therapeutic DLI after allo-HCT, three initially presented with molecular relapse that preceded overt hematologic recurrence by several weeks. In contrast, in most patients within the therapeutic DLI group, molecular and hematologic relapse were detected almost simultaneously, leaving little or no window for preemptive intervention. These observations highlight the key role of disease kinetics in determining the feasibility of preemptive DLI.

DLI efficacy likely reflects both leukemic proliferative dynamics and susceptibility to GvL-mediated immune control. Consequently, the clinical outcome after DLI integrates both features, which may interact at the single-cell level. In our cohort, intensified MRD monitoring and preemptive DLI prevented overt relapse in 23 patients, suggesting that timely immune intervention can successfully maintain disease control in a substantial subset of cases.

The observed association between GvHD following DLI and improved survival, while not implying a causality, likely reflects a GvL effect. GvHD occurred more frequently following preemptive DLI (57%) than therapeutic DLI (14%). Overall, GvHD affected a minority of patients in the entire cohort (27%). Among affected patients, most cases were grade I–II acute or mild-to-moderate chronic GvHD, although clinically relevant grade III–IV acute manifestations were also observed. These findings indicate a heterogeneous spectrum of immune activation, supporting the interpretation that the observed survival association reflects graft-versus-leukemia activity accompanied by varying degrees of immune-mediated toxicity. This observation is consistent with findings from a large study reporting that mild GvHD was associated with improved OS [23]. In contrast, Schmid et al. reported inferior OS among patients who developed GvHD after DLI [22], which may be explained by a higher cumulative incidence of severe GvHD in their cohort compared to ours. To account for this effect, we adjusted for GvHD in our multivariate models and interpreted as a post-intervention clinical event and not as a time-dependent variable, as the aim was to describe its association with outcome.

Survival did not differ significantly between diagnostic subgroups, likely reflecting the limited number of patients with MDS or ALL. A trend toward improved survival in MDS patients was observed, consistent with Schroeder et al. [24], and warrants confirmation in larger cohorts. Conversely, outcomes following DLI were poorest in patients with ALL, in line with Collins et al. [10]. Exploratory case review showed that in rare instances, molecular relapse preceded clinical relapse by several weeks to months. In most patients, however, loss of chimerism or rising MRD coincided with clinical or hematological disease recurrence, indicating that the window for intervention is usually narrow.

Of note, time from transplantation to relapse emerged as an important prognostic factor, in line with earlier findings [25] suggesting that early relapse reflects more aggressive disease biology.

Overall, our findings align with existing literature emphasizing the benefit of early intervention in post-transplant relapse and contribute additional evidence supporting a survival advantage with preemptive DLI compared to the therapeutic administration of this adoptive immunotherapy. However, several limitations must be acknowledged. This study is limited by its retrospective, single-center design and the small sample size in certain subgroups, which may affect generalizability. The unequal group sizes may introduce imbalance, and the absence of a comparator cohort with less intensive surveillance precludes causal inference regarding monitoring strategies. Consequently, our results reflect outcomes observed within a clinical setting characterized by intensive molecular surveillance rather than defining optimal monitoring intervals. Prospective multicenter studies with standardized monitoring intervals are needed to validate the observed survival benefit of preemptive DLI and to better define risk-adapted surveillance and intervention strategies.

## Conclusion 

Preemptive administration of DLI at the stage of molecular relapse was associated with significantly improved survival after relapse following allo-HCT in patients with acute leukemia or MDS. The occurrence of GvHD after DLI may reflect a beneficial GvL effect. In our setting of monthly MRD monitoring, early detection and timely intervention enabled favorable survival outcomes, supporting the hypothesis that closer surveillance may provide clinical benefit and enable earlier, more effective intervention. Prospective multicenter studies are needed to validate these findings and to refine risk-adapted monitoring and intervention strategies

## Data Availability

The datasets generated and/or analyzed during the current study are not publicly available due to data protection regulations but are available from the corresponding author on reasonable request.
